# Evidence for 24-hour posture management: A scoping review

**DOI:** 10.1177/03080226221148414

**Published:** 2023-01-12

**Authors:** Lauren Julia Osborne, Rosemary Joan Gowran, Jackie Casey

**Affiliations:** 1 University of Hertfordshire, Hatfield, Hertfordshire, England; 2 Discipline Occupational Therapy, Faculty of Education and Health Sciences, School of Allied Health, Health Research Institute, Health Implementation Science and Technology, University of Limerick, Limerick, Ireland; 3 School of Health and Sports Science, University of the Sunshine Coast, Queensland, Australia; 4 Assisting Living and Learning (ALL) Institute Maynooth University, Maynooth, Co. Kildare, Ireland; 5 Advanced Practitioner Occupational Therapist-Specialised Seating, Regional Rehabilitation Engineering Centre, Belfast Health & Social Care Trust, Belfast, Northern Ireland; 6 Faculty of Health and Life Sciences, Oxford Brookes University, Oxford, UK

**Keywords:** 24-hour postural management, assistive technology, postural care, national guidelines

## Abstract

**Introduction:**

People with complex physical disabilities unable to change their position independently are at risk of developing postural deformities and secondary complications. 24-hour posture management is needed to protect body structure. With inconsistencies in current service provision, this research aimed to scope the evidence for a 24-hour posture management approach.

**Method:**

A scoping review was conducted using four health and social science databases. Inclusion and exclusion criteria were applied; further papers were included through citation chaining.

**Results:**

The evidence for 24-hour posture management was often low quality due to the complications of completing robust research studies in this complex specialty. However, many professionals in the field agree that a 24-hour approach to postural care is essential.

**Conclusion:**

There is a need for clear national policy and guidance relating to postural care and scope for development of dedicated posture management services. Current NHS service provision is variable and inconsistent. Lack of postural care is a safeguarding and human rights issue. Specialist training and research in postural care within the Occupational Therapy profession is required to raise awareness of the role Occupational Therapists can play in preventing postural deformities and other secondary complications through providing good postural care.

## Introduction

For people with complex physical and sensory disabilities, who are unable to independently change their body position, posture and positioning become a very important issue. Their body structures are influenced by both postural alignment and the forces of gravity, which in turn affect body functions and the ability to participate in everyday activities. Posture is defined as the alignment of body segments relative to each other (Casey et al., 2020; Stinson et al., 2021). If an individual adopts an asymmetrical position, is not able to independently change position and is submitted to the forces of gravity they can develop secondary complications such as tissue damage, muscle contractures, pain and discomfort, constipation and infections (Pope, 2007); characteristics which are typically not a direct consequence of the diagnosed impairment (Pope, 2007). Often the body structures distort because of an inability to change position and an asymmetric posture, coupled with the influence of gravitational forces, which compress and restrict the function of the internal organs. If left uncorrected, this can lead to further health and participation complications and even premature death (Changing our Lives, 2018; Clayton et al., 2017).

To reduce the negative effects of gravity and avoid the development of such complications, ‘24-hour posture management’ is regarded as necessary (Hill and Goldsmith, 2010) by many therapists. Farley et al. (2003) define 24-hour postural management as utilisation of a range of interventions to reduce postural asymmetry and improve function, and includes all lying, sitting and standing positions that occur within any 24-hour period. Assessment of posture throughout the day and night needs to be examined within a multi-disciplinary framework to ensure appropriate provision of postural advice and supports (Castle et al., 2014; Hill and Goldsmith, 2009; Wright et al., 2010) that match the needs of both the individual and their caregivers. Techniques include the use of lying supports, standing supports and specialised seating (Agustsson and Jonsdottir, 2018) to sustain comfort and symmetry across multiple orientations that a person can spend significant lengths of time over a 24-hour period. If posture is not examined collectively in each orientation of sitting, lying and standing, it can be counterproductive to the provision of postural supports in only one orientation.

From an Occupational Therapy perspective, taking a ‘whole-person approach’ to support both mental and physical health and wellbeing is essential to enable individuals the opportunity to achieve their full potential (RCOT, 2019). Therefore, it is important for the profession to consider posture management as a pre-requisite (Agustsson and Jonsdottir, 2018) and fundamental to occupational performance. For example, if an individual is unable to maintain sitting balance because their trunk is not stable, their arms will naturally seek to provide the stability required; leaving their hands unavailable to engage in task performance. They may uneconomically use energy attempting to maintain an upright position, rather than engaging in an activity. Optimal postural care often relies on therapists’ knowledge and experience (Humphreys and Pountney, 2006; Pountney et al., 2002) leading to a postcode lottery of expertise, funding and timely provision of appropriate 24-hour postural management intervention.

In the United Kingdom, NHS provision of postural management assistive technology is ad hoc due to complicated and fragmented commissioning procedures and funding across varying models of postural management services (Aldersea, 1999; Darzi, 2018). Integration of these services as well as a joined-up approach to commissioning has long been recommended to improve efficiency and cost-effectiveness of equipment provision for posture management (Aldersea, 1999). Today, wheelchair services are well-established at meeting mobility and postural needs within wheeled mobility, but often there is no consideration given to the remainder of the 24-hour period, including no provision for static seating. Often, persons who need specialist static seating provision outside of a wheelchair, or who do not meet the criteria for a wheelchair, will fall into a gap in service provision with their needs going unmet. In these instances, individuals and families are left to purchase static seating independently, which can often lead to expensive mistakes of purchasing ill-fitting or inappropriate seating (Collins, 2001).

There is growing evidence that supports the notion that positioning in lying has a direct relationship on the success of postural alignment in sitting. Many individuals spend short periods of time in a wheelchair that has been custom made for their complex needs and frequently have no provision to address their postural needs outside of the wheelchair. There is often no funding for alternative seating or lying supports. Provision is patchy and inconsistent throughout the UK; it is not typically coordinated to ensure that postural supports work in harmony to cover the whole 24-hour period.

Failure to provide appropriate wheelchair seating and static seating leads to individuals being unable to reach their occupational potential and often not able to participate in life as an equal citizen (Gowran, 2013). At this point, posture management provision becomes an issue of safeguarding and human rights as it is seen as ‘a pre-requisite for survival and personal mobility’ (Gowran et al., 2020: 9). Therefore, the primary objective of this study is to examine the evidence for 24-hour posture management. Secondary objectives were to explore the current provision of postural care and consider recommendations that can improve postural management practice within the Occupational Therapy profession in the UK.

## Method

A scoping review methodology was used to synthesise the breadth of available literature and study designs employed. This method of scoping an emerging topic area that is broad, ensures that all available literature is included, including policy as well as intervention and exploratory research papers. Munn et al. (2018) suggested that scoping reviews offer a different technique for scrutinising theories and could therefore be more beneficial for practice development. As the aim of this study is to explore the use and merits of 24-hour posture management to inform practice, it is more appropriate to adopt a scoping method that captures rationale from across a range of sources.

Further, Anderson et al. (2008) stated that scoping studies are a crucial research methodology for guiding policy makers and can provide the opportunity to develop further research, policy and evidence-based practice (Colquhoun et al., 2014). Global literature was included since this is an emerging topic with patchy provision in the UK. It was felt there could be potential learning from the international community for implementing 24-hour posture management that might be relevant to the development of recommendations for enhanced practice within the UK.

This scoping review was structured using the PRISMA (Preferred Reporting Items for Systematic review and Meta Analyses) guidelines. The concept of posture management continues to evolve, so published research studies, policies, clinical frameworks and emerging unpublished studies and reports were included in the search. The literature review conducted by Farley et al. (2003) was used as the starting point for this review examining the evidence since 2003 for posture management interventions.

Relevant scientific literature was identified using four electronic databases: CINAHL, Psych INFO, Web of Science, and Medline covering the range of scientific literature published in educational, medical, psychological and social sciences journals. An initial search of PubMed was used to test out the preliminary search terms. A PICO framework was used to identify a list of 33 keywords based on the authors’ (LO and JC) experience of the field relating to posture and types of posture management devices, such as ‘custom moulded seating’. An additional 21 keywords describing postural deformities, such as ‘contracture’ and ‘hip subluxation’ were also identified (Table 1).

**Table 1.** PICO framework.

Population	Any person with complex and/or multiple disabilities, which result in the person being unable to independently transfer their weight, reposition themselves or move out of their base of support and back again without assistance.
	Any person with postural asymmetries and/or contractures.
	All diagnoses included.
	All ages included.
Intervention	24-hour Posture Management Programme
	Provision of wheelchairs, postural armchairs, static seating, sleep systems, night-time positioning systems and/or standing frames for the use of 24-hour posture management.
Context	A person’s own home.
	Residential and nursing homes.
	Schools.
Outcome	Reduction of postural deformities.
	Reduction of pressure ulcers.
	Improved pain, breathing, digestion, interaction, swallow, quality of life.

Each of the keywords listed under ‘posture management’ were searched using the Boolean operator ‘OR’ to capture all possible terms; this yielded 101,434 hits. These were then searched in association with the keywords against ‘postural deformities’ using the Boolean operator ‘AND’, yielding a total of 4387 hits. The Boolean phrase ‘AND’ was used to combine the search results, resulting in only five papers. Therefore, it was decided to remove the second column of postural deformity terms and widen the search by concentrating on the posture management keywords; using the disability and diagnosis terms did not enhance the search strategy. As it is an emerging topic, scoping the breadth of the literature, including such refined terms narrowed the search results. Research that is completed on this topic is catalogued using the main heading of postural terms, rather than being diagnosis-specific. This also ensured that there was no bias towards one particular diagnosis, as postural deformity can affect anyone who is unable to move their position independently and is not determined by their medical diagnosis.

However, with a result of over 100,000 papers, this needed to be refined. In consultation with the health librarians all generic terms such as ‘chair’ and ‘position*’ were removed as these were felt to be too broad. Papers not relevant to the research question were excluded. The final search terms were ‘posture management’, ‘night-time positioning’, ‘seating assessment’ and ‘postural seating’; these were truncated to include similar terms such as ‘posture’ and ‘postural’. The grey literature was retrieved via a Google search engine using the search terms and names of known authors in the field. They were then reviewed and scanned in the same way as the retrieved published peer-reviewed literature and the same inclusion/exclusion criteria were applied.

Titles and abstracts were independently reviewed by two authors (LO and JC), then full texts obtained if the abstract appeared relevant or if it seemed unclear. The following inclusion and exclusion criteria were applied to focus the search into meaningful results.

## Inclusion

- Scientific and grey literature relating to 24-hour posture management
- Literature relating to use of wheelchairs, specialised seating, night-time positioning and standing frame use within the context of 24-hour posture management
- Literature worldwide
- Written in English language only
- Dated from 2003 to 2019

## Exclusion

- Non-English language literature
- Dated pre-2003
- Literature detailing product testing or development
- Literature relating to wheelchairs, specialist seating, night-time positioning and standing frames specifically, without applying the context of 24-hour posture management.

As the papers from each database were reviewed, so too were their reference lists; a method referred to as ‘citation chaining’ (Greenhalgh, 2010). Typically, a scoping review does not critically appraise the evidence presented (Colquhoun et al., 2010; Peters et al., 2015). However, it was felt necessary to comment on the strength of the research available to demonstrate the challenges of conducting research in this field, and to consider the reasons for this and hence the importance of considering other types of literature.

The papers were read multiple times and the primary author was fully immersed in the literature. A simple categorisation system by Wallace and Wray (2006), cited in Aveyard (2014) was used to categorise the literature type and then an interpretive phenomenological approach was used to identify common themes arising across all of the literature.

**1. Theoretical literature** – developing theories through examining evidence.
**2. Research literature** – reporting a specific enquiry into a topic or treatment method.
**3. Practice literature** – expert opinion, debate, discussion papers from industry experts.
**4. Policy literature** – national or local policies and guidelines that inform practice.
(Wallace and Wray, 2006 cited in Aveyard, 2014: 44).

## Results

**Figure 1.**
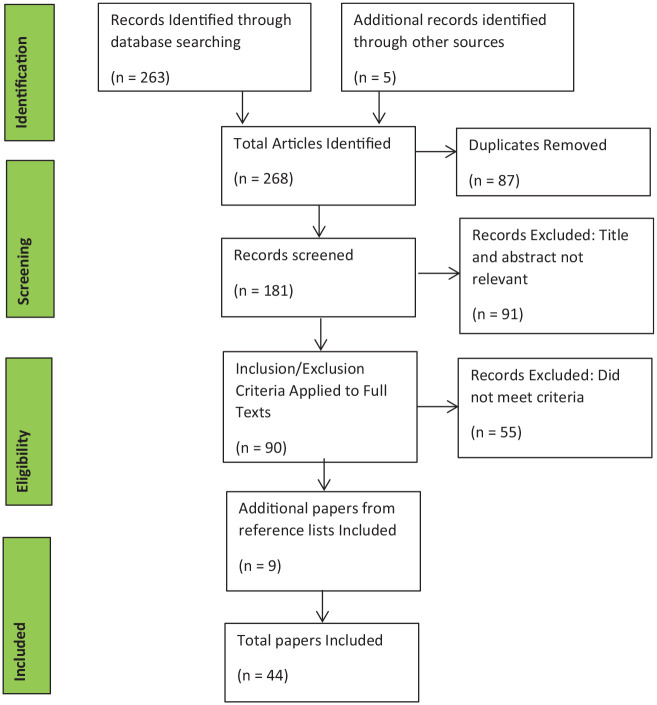
PRISMA Diagram showing search results.

A total of 268 papers were retrieved, with 44 meeting the inclusion criteria. Of these, half were centred around a view of 24-hour posture management, or posture management in general, 10 were focused on lying postures and night-time positioning, 8 on sitting posture and seating assessment and 4 on standing programmes (see Figure 1).

### Types of papers

Of the 44 papers included, 4 were classified as theoretical literature, 25 as research literature, 13 as practice literature, and 2 as policy literature. See Table 2.

**Table 2.** Included papers: Categorised according to Literature category.

Paper	Literature Type
Aburto, N. (2016) Sleep System Service Evaluation. *Hounslow Wheelchair and Posture Management Service.* NHS Hounslow and Richmond Community Healthcare Trust.	Research
Birth Defects Foundation Newlife. (2007) It’s not too much to ask: *Campaign Report*: Cannock: BDF Newlife.	Policy
Blake, S. F., Logan, S., Humphreys, G., Matthews, J., Rogers, M., Thompson-Coon, J., Wyatt, K. and Morris, C. (2015) Sleep positioning systems for children with cerebral palsy. *The Cochrane database of systematic reviews*, (11), pp. CD009257.	Research
Casey, J., Hoffman, L., Hutson, J. and Kittelson-Aldred, T. (2019) Supporting the occupation of sleep through night-time positioning equipment. *SIS Quarterly Practice Connections*, 4 (2), pp. 7-9.	Practice
Castle, D., Stubbs, B., Clayton, S. and Soundy, A. (2014) A 24-hour postural care service: Views, understanding and training needs of referring multidisciplinary staff. *International Journal of Therapy and Rehabilitation*, 21(3), pp. 132-139.	Research
Clayton, S. (2013) Living Local Postural Care Project Evaluation. *Postural Care CIC*.	Practice
Collins, F. (2008) An essential guide to managing seated patients in the community. *British Journal of Community Nursing*, 13(3), pp. 39-40.	Practice
Crawford, S. and Curran, A. (2014) 24 Hour Postural Management for Community Dwelling Adults with Learning Disabilities. *The Journal of Posture and Mobility Group*, 31, pp. 15-19.	Research
Crawford, S. and Stinson, M. (2015) Management of 24-h-Body Positioning. In: Söderback, I. (ed.) *International Handbook of Occupational Therapy Interventions*. Cham: Springer International Publishing, pp. 189-203.	Theoretical
Daly, G. (2017) Prevention and postural management of residents in care homes. *RCOT Annual Conference*, pp. 109.	Practice
Daly, O., Casey, J. and Martin, S. (2013) The effectiveness of specialist seating provision for nursing home residents. *A report for Seating Matters Ltd*. Knowledge Transfer Partnership (KTP) Sept 2011-Sept 2013.	Research
Day, E. and Lockwood, D. (2012) The impact of posture management on two adults with learning disabilities. *RCOT Annual Conference*, pp. 49.	Practice
Farley, R., Clark, J., Davidson, C., Evans, G., Maclennan, K., Michael, S., Morrow, M. and Thorpe, S. (2003) What is the evidence for the effectiveness of postural management? *International Journal of Therapy and Rehabilitation.* Vol 10, No 10. Pg 449-455.	Research
Fields, F. & McGrath, M. (2008) 24 hour postural management: a new direction in therapeutic care. *Physiotherapy Ireland*, 2008 Jun; 29(1): 75-75.	Practice
Gmelig Meyling, C., Ketelaar, M., Kuijper, M., Voorman, J. and Buizer, A. (2017) Postural management to reduce or prevent hip migration in children with cerebral palsy: a systematic review. *Developmental Medicine & Child Neurology*. Pediatric Physical Therapy vol. 30,2: 82-91.	Research
Goodwin, J., Lecouturier, J., Basu, A., Colver, A., Crombie, S., Smith, J., Howel, D., McColl, E., Parr, J. R., Kolehmainen, N., Roberts, A., Miller, K. and Cadwgan, J. (2018a) Standing frames for children with cerebral palsy: a mixed-methods feasibility study. *NIHR Health Technology Assessment.* Vol 22, Issue 50.	Research
Goodwin, J., Lecouturier, J., Crombie, S., Smith, J., Basu, A., Colver, A., Kolehmainen, N., Parr, J. R., Howel, D., McColl, E., Roberts, A., Miller, K. and Cadwgan, J. (2018b) Understanding frames: A qualitative study of young people's experiences of using standing frames as part of postural management for cerebral palsy. *Child: care, health and development*, 44(2), pp. 203-211.	Research
Goodwin, J., Lecouturier, J., Smith, J., Crombie, S., Basu, A., Parr, J. R., Howel, D., McColl, E., Roberts, A., Miller, K. and Cadwgan, J. (2019) Understanding frames: A qualitative exploration of standing frame use for young people with cerebral palsy in educational settings. *Child: Care, Health and Development*, 45(3), pp. 433-439.	Research
Gough, M. (2009) Continuous postural management and the prevention of deformity in children with cerebral palsy: An appraisal. *Developmental Medicine & Child Neurology*, Vol 51(2), Feb, 2009 pp. 105-110.	Theoretical
Hill, C. M., Parker, R. C., Allen, P., Paul, A. and Padoa, K. A. (2009) Sleep quality and respiratory function in children with severe cerebral palsy using night-time postural equipment: a pilot study. *Acta Paediatrica*, 98, pp. 1809-1814.	Research
Hill, S. (2011) A one year postural care training programme for the workforce supporting the needs of those with complex and continuing healthcare needs: Project Evaluation. *Postural Care CIC*.	Practice
Hill, S. and Goldsmith, J. (2010) Biomechanics and prevention of body shape distortion. *Tizard Learning Disability Review*, 15(2), pp. 15-32.	Theoretical
Hill, S. and Goldsmith, L. (2009) Mobility, Posture and Comfort. In: Pawlyn, J. and Carnaby, S. (eds.) (2009) *Profound Intellectual and Multiple Disabilities: 9781444301533*: Wiley-Blackwell Publishing, Chichester, pp 328-346.	Theoretical
Hotham, S. et al. (2017) A study into the effectiveness of a postural care training programme aimed at improving knowledge, understanding and confidence in parents and school staff. *Child: Care, Health and Development*, Vol 43(5), Sep, 2017 pp. 743-751.	Research
Humphreys, G. and Pountney, T. (2006) The development and implementation of an integrated care pathway for 24-hour postural management: a study of the views of staff and carers. *Physiotherapy*, 92(4), pp. 233-239.	Research
Humphreys, G., King, T., Jex, J., Rogers, M., Blake, S., Thompson-Coon, J. and Morris, C. (2019) Sleep positioning systems for children and adults with a neurodisability: A systematic review. *British Journal of Occupational Therapy*, 82(1), pp. 5-14.	Research
Hutton, E. and Coxon, K. (2011) ‘Posture for Learning’: meeting the postural care needs of children with physical disabilities in mainstream primary schools in England – a research into practice exploratory study. *Disability and Rehabilitation*, 33:19-20. 1912-1914.	Research
Innocente, R. (2014) Night-time positioning equipment: A review of practices. *New Zealand Journal of Occupational Therapy*, 61(1), pp. 13-20.	Practice
Jones, C., Underhill, M. and Baylis, M. (2015) Service transformation through integration: specialist seating model for children. *RCOT Annual Conference*, pp. 77.	Practice
Kent, F. (2015) 24-hour postural management: all about seating 1 – practical considerations. *National Association of Equipment Providers*, Oct 2015; 98-103.	Practice
Lansdown, K. and Dawson, K. (2019) Are you sitting comfortably: An evaluation of a postural management masterclass for occupational therapy staff. *RCOT Annual Conference*, pp. 72.	Practice
Lyons, E. et al. (2017) An exploration of comfort and discomfort amongst children and young people with intellectual disabilities who depend on postural management equipment. *Journal of Applied Research in Intellectual Disabilities*, Vol 30(4), Jul, 2017 pp. 727-742.	Research
Macias-Merlo, L. et al. (2016) Effects of the standing program with hip abduction on hip acetabular development in children with spastic diplegia cerebral palsy. *Disability And Rehabilitation* Volume: 38 Issue: 11 Pages: 1075-1081 Published: MAY 21 2016	Research
Maher, C. et al. (2011) Factors influencing postural management for children with cerebral palsy in the special school setting. *Disability and Rehabilitation: An International, Multidisciplinary Journal*, Vol 33(2), 2011 pp. 146-158.	Research
McDonald, R., Surtees, R. and Wirz, S. (2004) The International Classification of Functioning, Disability and Health provides a Model for Adaptive Seating Interventions for Children with Cerebral Palsy. *British Journal of Occupational Therapy*, 67(7):293-302.	Practice
O'Connor, B., Boyd, R. N. and Shields, N. (2006) A systematic review of postural management of hip displacement in children with cerebral palsy. *Developmental Medicine & Child Neurology*, (48), pp. 42-43.	Research
Owens, K. & Daly, G. (2016) A study into the effectiveness of 24 hour postural care in the management of contractures in Care Homes. Middlesborough Council.	Research
Polak, F., Clift, M. and Clift, L. (2009) *Buyers' Guide. Night-time postural management equipment for children CEP08030.*: London: NHS Centre for Evidence-based Purchasing.	Policy
Pountney, T. et al. (2009) Hip subluxation and dislocation in cerebral palsy – a prospective study on the effectiveness of postural management programmes. Physiotherapy Research International: The Journal for Researchers And Clinicians In Physical Therapy, 2009 Jun; Vol. 14 (2), pp. 116-27.	Research
Robertson, J. et al. (2018) Postural care for people with intellectual disabilities and severely impaired motor function: A scoping review. *Research in Intellectual Disabilities*, Vol 31(Suppl 1), Jan, 2018 pp. 11-28.	Research
Seabrook, R. (2017) Specialist seating - a model to increase efficiencies in provision and meet a growing demand. *RCOT Annual Conference*, pp. 114.	Practice
Stephens, M., Bartley, C. and Priestley, C. (2018) Night-time Positioning for Care Home Residents. [Online} URL: https://pdfs.semanticscholar.org/fae9/f3c7670c8226384c266aa5562f0e24249d49.pdf (Accessed 18/01/20).	Research
Wright, C., Casey, J. and Porter-Armstrong, A. (2010) Establishing best practice in seating assessment for children with physical disabilities using qualitative methodologies. *Disability and Rehabilitation: Assistive Technology*, 5(1), pp. 34-47.	Research
Wynn, N. and Wickham, J. (2009) Night-time positioning for children with postural needs: What is the evidence to inform best practice? *The British Journal of Occupational Therapy.*, 40(12), pp. 543.	Research

### Study design

There were no RCTs and only one quasi-experimental design amongst the included research papers, supporting the view these research methodologies are not appropriate for this population and clinical field (see Table 3).

**Table 3.** Classification of scientific papers by research design.

Research design	No of papers	Research design	No of papers	Research design	No of papers
Action Research	2	Evaluation	2	Scoping Review	1
Case Study	2	Exploratory	4	Sequential	2
Cohort	1	Observation	1	Systematic Review	6
Comparison	1	Pilot Study	1		
Cross-sectional Descriptive	1	Quasi-Experimental	1		

Most papers were systematic literature reviews, concluding that the research available was weak in design and of low quality (Blake et al., 2015; Gmelig Meyling et al., 2017; Humphreys et al., 2019; O’Connor et al., 2006) and stipulated that further research was recommended such as development of a consensus statement of expert opinion and regular publishing of case studies from clinical practice (Farley et al., 2003; Wynn and Wickham, 2009).

Whilst the evidence body is small, it is growing and does appear to support 24-hour posture management (Wynn and Wickham, 2009). The systematic review by Blake et al. (2015) also noted that many papers included in their review were not indexed on electronic databases and were retrieved through directly contacting manufacturers and authors, illustrating that literature on this topic is hard to locate. An explanation for this may be due to strict submission criteria and that many journals will not publish research with lower levels of research design.

Of the 13 papers classified as practice literature, 6 were conference abstracts of presentations given by clinicians working within the posture management field. Half of those directly stated that Occupational Therapists have a direct role in assessing and delivering posture management (Daly, 2017; Day and Lockwood, 2012; Lansdown and Dawson, 2019). Whilst the other half presented their own development of multidisciplinary working towards a posture management service in their geographical area (Fields and McGrath, 2008; Jones et al., 2015; Seabrook, 2017). Four of the practice papers were from clinicians writing to review an area of their practice or providing their own guidance from experience within the field (Casey et al., 2019; Collins, 2008; Innocente, 2014; McDonald et al., 2004). The remaining three were projects from the specialist interest groups National Association of Equipment Providers and Postural Care CIC, exploring relevant evidence, policy and guidelines, then making recommendations for future service provision (Clayton, 2013; Hill, 2011; Kent, 2015).

This demonstrates that clinicians on the front line have recognised a need for postural management and are beginning to implement interventions and seek out an evidence base for this. In addition, this suggests that they are searching for policy or guidance to steer them and to share their findings with peers, indicating that posture management is a tangible issue that is increasingly part of standard Occupational Therapy practice.

The team discussed the themes and further analysis condensed these into four over-arching themes which were then linked back to the primary objective (see Table 4):

1. Education on posture management
2. Posture management across the three primary positions
3. Impact on occupational performance and participation
4. Service provision

**Table 4.** Included papers: Categorised according to emerging themes.

Paper	Service provision	Education	Posture management	Occupational Performance
Aburto (2016)	X			
Birth Defects Foundation Newlife (2007)	X			
Castle et al. (2014)		X		
Clayton (2013)			X	
Collins (2008)			X	
Crawford and Curran (2014)		X		
Crawford and Stinson (2015)		X		
Daly (2017)			X	X
Daly et al. (2013)			X	
Day and Lockwood (2012)			X	X
Fields and McGrath (2008)	X			
Goodwin et al. (2018)		X	X	
Hill et al. (2009)			X	
Hill (2011)			X	
Hill and Goldsmith (2010)			X	
Hutton and Coxon (2011)		X		
Innocente (2014)			X	
Jones et al. (2015)	X			
Kent (2015)			X	
Lansdown and Dawson (2019)	X			
McDonald et al. (2004)	X			
Polak et al. (2009)			X	
Seabrook (2017)	X			
Stephens et al. (2018)			X	
Wright et al. (2010)			X	
Wynn and Wickham (2009)			X	

#### Education on posture management

Literature identified similar definitions of posture management as the use of a range of techniques that can be used to preserve body shape to prevent secondary complications, promoting a symmetrical body shape and enhance function and participation, whilst recognising that its use is mainly directed at individuals who are unable to change their position independently (Castle et al., 2014; Crawford and Curran, 2014; Crawford and Stinson, 2015). These benefits to health and wellbeing will have an impact on the individual’s quality of life.

There was also a clear, defined need for 24-hour posture management, recognising that many of the secondary complications that can occur due to lack of posture management are avoidable and therefore, clinicians have a duty of care to prevent their development (Castle et al., 2014).

In addition, there is a need for further training for healthcare professionals in prescribing postural care techniques and associated devices as well as for families and carers at how best to apply those techniques and devices. Castle et al. (2014) found that most staff within the multidisciplinary team had no understanding of the need for postural management and they did not have any assessment tool for identifying need. Instilling staff and family knowledge and understanding of the need for posture management and the benefits it can produce, will help to alleviate any fear or anxieties of not applying postural supports appropriately and reduce the risk of causing harm (Goodwin et al., 2019; Hutton and Coxon, 2011).

#### Posture management across the three primary positions

**Posture management in lying:** Posture management in a lying position is a crucial part of 24-hour posture management, owing to the length of time spent in bed (Clayton, 2013; Hill, 2011; Hill et al., 2009; Hill and Goldsmith, 2010; Innocente, 2014; Polak et al. 2009; Wynn and Wickham, 2009) and the body being more receptive to specific positioning when muscle tone is more relaxed (Polak et al., 2009; Stephens et al., 2018). Positioning during the day can be influenced by the position adopted overnight (Polak et al., 2009). Symmetrical supine lying is reported to be the optimum position to aid balanced functioning of the muscles (Hill and Goldsmith, 2010; Polak et al., 2009).

**Posture management in sitting:** Seating is only one aspect of 24-hour posture management and therefore should not be viewed exclusively without considering the other orientations of posture (Kent, 2015). People sit for several hours per day (Daly et al., 2013; Wright et al., 2010), so seating must be correctly fitting and provide adequate pressure distribution (Collins, 2008). One client group who are particularly in need of specialist seating are older people living in care homes. Despite demonstrated benefits, there is a lack of clinicians with specialised seating knowledge and very little advice in national guidelines to inform the assessment and prescription process, with no national care pathway for seating (Collins, 2008; Wright et al., 2010).

**Posture management in standing:** The benefits of standing as part of a posture management programme have been reported as improved bone mineral density, hip stability, joint range of movement, reduced pain, improved muscle stretch and increased participation (Goodwin et al., 2018).

#### Impact on occupational performance and participation

It is recognised that provision of appropriate postural seating can increase participation in activities through improving the accessibility and awareness of the environment, as well as enabling individuals to be more functional in a better supported position (Daly, 2017). The involvement of Occupational Therapists in the provision of seating offers a distinctive approach in how positioning affects a person’s ability to participate (Day and Lockwood, 2012). Sleep is often overlooked as an occupation, plus having good sleep can have additional positive benefits to daytime occupational engagement.

#### Service provision

McDonald et al. (2004) referred to the Audit Commission (2000) report ‘Fully Equipped’ as emphasising poor strategic and operational methods for delivery of mobility services. The disbanding of healthcare services has subsequently resulted in a postcode lottery of service provision, with much confusion not only amongst members of the public, but also health and social care professionals about where and what funding is provided, and often with insufficient budgets allocated (Birth Defects Foundation Newlife, 2007). The lack of providing adequate individualised postural support devices, whether it is an armchair, night-time positioning system or a standing frame can lead to more complex postural deformities developing and therefore ultimately present a higher cost, requiring more specialised care and equipment for long-term care plans, which could be prevented. Also, adopting a pro-active intervention at the outset would often reduce the likelihood of the individual having pain and developing preventable secondary complications (Daly, 2017). The numbers of people requiring posture management equipment are a relatively small proportion of an overall clinical group. However, their needs are more complex and therefore use a greater proportion of the limited resources (Aburto, 2016). It could be assumed that if their postural needs were addressed early before they deteriorate and become non-reducible requiring complex care, more resources would be available to share. On a positive note, dedicated posture management services are starting to emerge in different areas across the UK (Aburto, 2016; Crawford and Curran, 2014; Fields and McGrath, 2008; Robertson et al., 2018; Seabrook, 2017). Further, several clinicians are now presenting on this topic at conferences (Daly, 2017; Day and Lockwood, 2012; Fields and McGrath, 2008; Jones et al., 2015; Lansdown and Dawson, 2019; Seabrook, 2017), which shows there is some innovation of service development across the professional field. With wheelchair services in much of the UK often being tendered to private providers, competition for contracts could be an opportunity to drive pioneering service provision to offer an advantage over competitors (Daly et al., 2013). This could present an opportunity for posture management to become embedded into wheelchair services and enable a more rounded and coordinated 24-hour postural management approach to be adopted.

## Discussion and recommendations

There currently is no global interest group or platform for influencing postural care on a wider scale. Therefore, initial recommendations are tailored to improve the provision of postural care within UK practice. Many papers identify a lack of robust evidence for posture management techniques and interventions, all recognising there was a lack of randomised control trials (RCTs). Although often perceived as the ‘gold standard’ of research design, complex rehabilitation does not easily lend itself to RCTs. It would be unethical to withhold posture management interventions when the harmful effect of gravity on body-structures is well recognised. Further, blinding of the researchers on whether postural supports are used or not would be difficult depending upon the type of outcome measures employed; thereby making it very difficult to have a treatment group versus control group. Much of the literature cited in this scoping review is based on specialist clinical knowledge and expertise gained from real-life experience of working with the complex population who require posture management.

There are multiple authors from this field with similar conclusions; namely that 24-hour posture management can prevent the development of secondary complications, pressure ulcer development and improve health and wellbeing outcomes so that people are able to engage in occupation to live a more meaningful and comfortable life. Considering Humphreys et al.’s (2019) conclusions, a consensus of expert opinion on the subject would be beneficial to drive this agenda forwards into development of policy to guide assessment of need and provision of intervention. Therefore, it is recommended that a consensus paper on 24-hour posture management is produced, leading to development of national policy and guidance for Clinicians, individuals and their carers in the delivery of postural care for all people that have complex physical needs and who are at risk to the effects of gravity on their posture. It is important to recognise that this is an issue that does not only affect one client group and this work presents an opportunity to guide the creation and delivery of postural care services for all who need it. At present, there are guidelines around pressure ulcer prevention (NICE, 2014) and spasticity management in under 19’s (NICE, 2012) but these do not go far enough to recognise that habitual asymmetric postures can affect any person with complex physical disabilities, all age groups, all positions, and those unable to change their position independently.

Funding is a key barrier for preventing access to postural care. According to the Human Rights Act (1998), everyone has a ‘right to life’ and the secondary complications caused by poor posture management have been presented as a cause of premature death. Therefore, access to postural management services is a human right. The Audit Commission (2002: 4) stated that commissioning was a real problem for the provision of equipment and that ‘service commissioners and providers generally have no idea about the underlying level of demand for equipment services. Unmet need represents a major cause of social exclusion’. This is reflected in the findings from the Birth Defects Foundation Newlife (2007) campaign report; and in the Guidance for Postural Care and People with Learning Disabilities, Public Health England (2018), it is stated ‘It is recommended that clinical commissioning groups should ensure they commission expert, preventative services including proactive postural care support’. It has been reported that current service provision is patchy and inconsistent. With postural care being so devolved and postural supports for the three orientations of lying, sitting and standing being provided in isolation, or not at all, the current provision falls far short of the joined-up service that is advocated. To address this, development of a dedicated posture management service where all postural care needs and equipment is assessed for and provided as part of a holistic, 24-hour approach is recommended. This provides an opportunity to reduce duplication and ensure that all prescribed devices and recommendations work collectively.

For postural care to be recognised and delivered appropriately, it is proposed that universities in the UK and Ireland must include posture management training for undergraduate students and that training is multidisciplinary (Collins, 2012, cited in Kent, 2015). Additionally, training is required for families and carers of people who need postural care to ensure they are competent and confident to transfer and position the individual using the equipment so that it is used effectively and does not cause more harm (Blake et al., 2015; Hill et al., 2009; Polak et al., 2009). Posture management equipment is often very expensive and therefore, not investing in training families and carers in how to use it effectively, is a waste of resources and can have a negative effect for the individual through insufficient or incorrect posture management (Polak et al., 2009).

A limitation of this scoping review could be the exclusion of standing for which there is already an extensive body of published literature, particularly within physiotherapy. The authors recognise that standing is a vital strand of 24-hour postural care programmes and it is important that physiotherapy colleagues are part of the overall pathway. Another limitation is, there was no analysis on the complexity of postural supports used. For example, night-time positioning did not differentiate between using simple off-the-shelf pillows and wedges or a custom-moulded system. It was also acknowledged that none of the literature explored the perspective of the person using the assistive devices, although some did comment on the carer’s perspective.

As highlighted by Blake et al. (2015), it is important to be mindful when reviewing literature in this field of any potential bias and ethical concerns regarding objectivity when manufacturers may have sponsored research or provided assistive technology devices.

## Conclusion

A scoping review was conducted to examine the evidence for 24-hour posture management, which is regarded as necessary to reduce the effects of postural asymmetries and gravity on people that are unable to change their position independently and prevent the development of postural deformities and pressure ulcers. Whilst the evidence for 24-hour posture management may not be considered robust or of sufficient research quality, there is evidence none-the-less that holds weight and is valuable in guiding future practice. There are clear health and wellbeing benefits from the provision of 24-hour posture management for people who are unable to change their position independently.

Current NHS service provision for posture management and commissioning is weak and insufficient. The resulting postcode lottery of service provision is inadequate (Birth Defects Foundation Newlife, 2007) and failure to provide adequate posture management can be considered a safeguarding issue and a breach of human rights (Gowran, 2020). Therefore, to advance the posture management agenda, we urgently need to develop national guidance and a posture care pathway informing future practice. There are opportunities for the development of a dedicated posture management service through the expansion of current wheelchair services to include the provision of night-time positioning equipment, standing frames and postural static seating.

**Key findings**

- Current NHS provision of posture management is inadequate, resulting in unmet needs.
- Research in this field is challenging; subsequently, greater credibility should be attributed to specialist expertise.
- National guidelines are needed.

**What the study has added**

This review identifies the need for best-practice clinical guidelines on 24-hour posture management; scope for dedicated posture management services and increased training throughout the Occupational Therapy profession on 24-hour postural management intervention.
